# Synthesis and characterization of 1,2,3,4-naphthalene and anthracene diimides

**DOI:** 10.3762/bjoc.20.155

**Published:** 2024-07-25

**Authors:** Adam D Bass, Daniela Castellanos, Xavier A Calicdan, Dennis D Cao

**Affiliations:** 1 Chemistry Department, Macalester College, 1600 Grand Avenue, Saint Paul, Minnesota 55105, United States

**Keywords:** electron acceptors, organic materials, polycyclic aromatic hydrocarbons

## Abstract

We report the synthesis and characterization of naphthalene and anthracene scaffolds end-capped by cyclic imides. The solid-state structures of the *N*-phenyl derivatives, determined by X-ray crystallography, reveal changes in packing preference based on the number of aromatic rings in the core. The optical and electronic properties of the title compounds compare favorably with other previously described isomers and expand the toolbox of electron-deficient aromatic compounds available to organic materials chemists.

## Introduction

**Figure 1:**
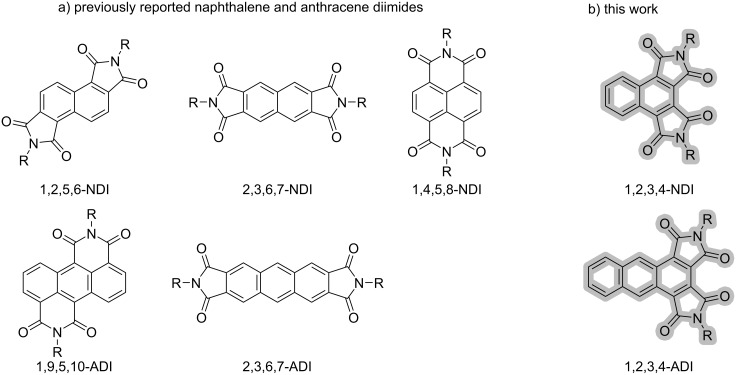
a) Structures of previously reported naphthalene and anthracene diimide isomers. b) The novel 1,2,3,4-naphthalene and -anthracene diimides reported here.

Aromatic diimides are ubiquitous molecular scaffolds that have served as the basis for robust polymers, supramolecular assemblies, and (opto)electronic materials. The vast majority of this research has focused on 1,2,4,5-benzene (pyromellitic), 1,4,5,8-naphthalene, and 3,4,9,10-perylene diimides. Beyond these, researchers have demonstrated that translocating the cyclic imides around the periphery of the aromatic core to yield different structural isomers is effective for producing interesting new materials. Ourselves and others have investigated 1,2,3,4-benzene diimide, also known as mellophanic diimide [1]**,** as a building block for heteroacenes [2–5] and polyimides [6–8]. The 1,2,5,6- [9–10] and 2,3,6,7-naphthalene diimides (NDIs) have been produced and utilized in electronically active polymers (Figure 1). The linear extension of 1,4,5,8-naphthalene diimide to produce tetracene [11] and hexacene [12] diimides, some with interesting properties such as near-IR absorption, has been achieved as well. Other efforts have demonstrated that anthracene diimides (ADIs) can be tuned to achieve decent electron mobilities in electronic settings [13–14]. Although there have been calculations conducted that suggest 6-membered cyclic imides are more compelling than 5-membered cyclic imides in organic electronic materials [15], the experimental observation of similar electron mobilities across different structural isomers of naphthalene and anthracene diimide [16] confirms the need to experimentally evaluate the unstudied isomers.

We became interested in the *cata*- (i.e. 1,2,3,4-) derivatization of aromatic scaffolds because it can be exploited to stabilize the longer (hetero)acenes. In contrast to *cata*-benzannulation, *cata*-imide-annulation does not perturb aromaticity patterns and further introduces inductive stabilization of frontier MO levels, which has enabled the production of n-type organic thin-film transistors from heteroacenes. Inspired by these results, we sought to demonstrate the preparation of all-carbon scaffolds, i.e., acenes, that are *cata*-annulated with cyclic imides. Here, we communicate the successful synthesis of 1,2,3,4-NDIs and -ADIs and the characterization of their physical properties.

## Results and Discussion

### Synthesis

**Scheme 1:**
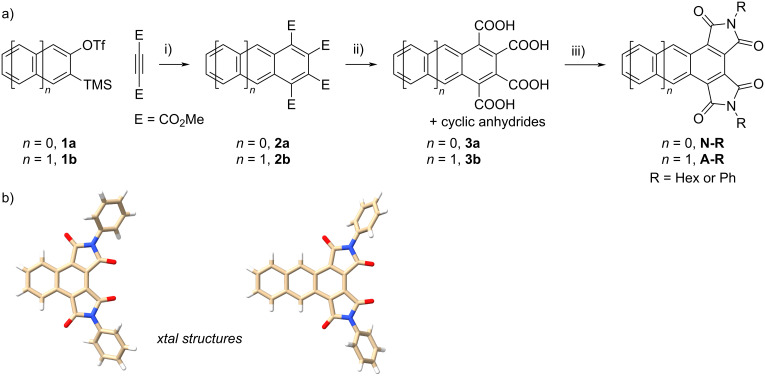
a) Synthesis of the 1,2,3,4-naphthalene and -anthracene diimides. Conditions: i) CsF/Pd_2_(dba)_3_/MeCN; ii) NaOH/H_2_O/THF, then HCl; iii) R-NH_2_/AcOH. b) X-ray crystal structures of **7-Ph** and **8-Ph**.

The synthesis of the title compounds is shown in Scheme 1. To obtain a naphthalene core with the requisite 1,2,3,4-tetracarbonyl derivatization pattern, we leveraged the cycloaddition of 1 equiv of aryne percursor **1** with 2 equiv of dimethyl acetylenedicarboxylate (DMAD). Although this [2 + 2 + 2] cycloaddition reactivity strategy has been reported under a variety of aryne generation conditions [17–19], in our hands we were only able to generate practical amounts of tetraesters **3** using the method reported by Peña et al. [18]. Hydrolysis of **3** with sodium hydroxide, followed by acidification with HCl, yielded a mixture of carboxylic acids and anhydrides **5**, as evidenced by ^1^H NMR spectroscopy (Figure S13 in Supporting Information File 1). Gratifyingly, purification of these mixtures was not necessary as they could be used directly for imidization. Heating **5** with hexylamine or aniline in refluxing acetic acid successfully led to the formation of the targeted aromatic diimides bearing either *N*-hexyl (**7-Hex**) or *N*-phenyl (**7-Ph**) substitutions in good yields. The same strategy was employed to create the imide-capped anthracenes **8-Hex** and **8-Ph**.

### Crystallography

**Figure 2:**
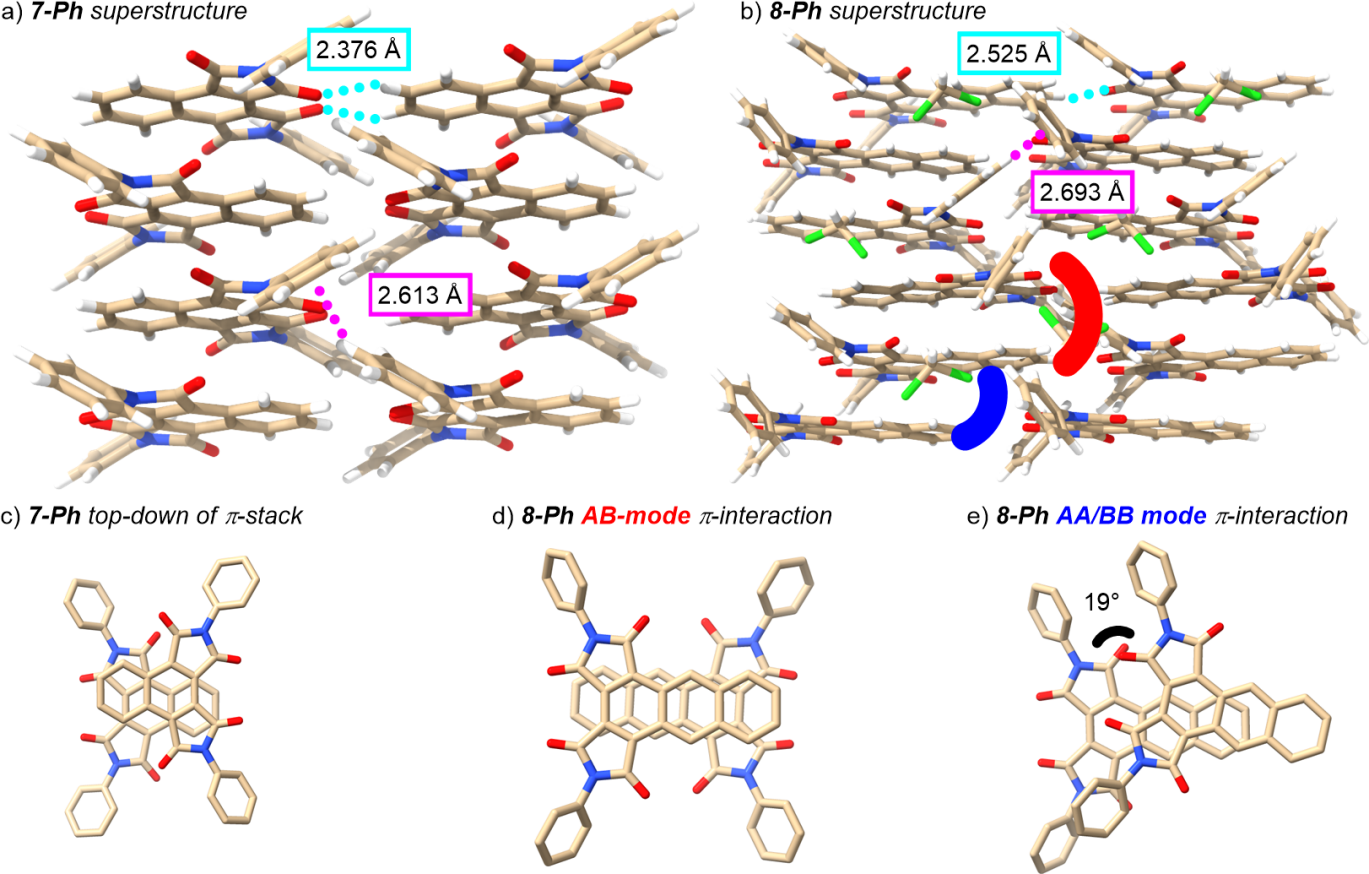
Superstructures for a) **7-Ph** and b) **8-Ph** as determined by X-ray crystallography. Representative C=O···H–C and C–H···π interactions are indicated in teal and magenta, respectively. Top-down views of π-stacking modes in c) **7-Ph** and d, e) **8-Ph**. Hydrogen atoms have been removed for clarity in c–e. Atom color code: C = tan, H = white, Cl = green, N = blue, and O = red.

Despite exhaustive efforts, we were unable to obtain single crystals of **7-Hex** and **8-Hex**; these compounds formed polycrystalline bundles that are fragile and insufficient for obtaining diffraction data. Fortunately, single crystals of the *N*-phenyl compounds were successfully grown by slow evaporation of CH_2_Cl_2_/MeOH solutions and characterized by X-ray crystallography. **7-Ph** crystallizes in the *Pbcn* space group into a solvent superstructure of π-stacked columns of **7-Ph**. The imide groups are pointed in alternating directions within a stack. While this may occur in part as a consequence of the steric demands of the *N*-phenyl groups, there are also C–H···π interactions between phenyl groups of adjacent π stacks, with the closest Ph centroid to H distance being 2.613 Å (Figure 2a). Additionally, the middle carbonyl oxygens are in short contact (2.376 Å) with the 7- and 8-H atoms of the naphthalene in the adjacent molecule (Figure 2a).

On the other hand, **8-Ph** crystallizes in the *Pbca* space group with two molecules of interest along with one molecule of CH_2_Cl_2_. Pairs of **8-Ph** molecules are π-stacked together with their imide groups oriented in opposing directions (Figure 2d) in a fashion analogous to that observed for **7-Ph** (Figure 2c). These pairs, however, are then infinitely packed such that adjacent **8-Ph** molecules are aligned in the same direction to create an AA–BB stacking pattern, unlike the more common A–B–A–B stacking pattern found for **7-Ph**. Additionally, it is worth observing that the π-interaction involving two **8-Ph** molecules pointed in the same nominal direction is not linearly aligned, but is instead twisted by 19°. This angle, likely enforced by the sterics of the phenyl groups, may be an interesting approach to inducing helical turns in supramolecular assemblies derived from the title compounds.

Despite this different packing mode within the stack, the interstack interactions exhibited by **8-Ph** are similar to those found in **7-Ph**. Although there are still observable C–H···π interactions and C=O···H–C interactions between stacks, they appear to be weaker, as evidenced by the longer interaction distances and interceding incorporation of CH_2_Cl_2_ (Figure 2b). Furthermore, in **8-Ph** the interstack C=O···H–C interaction is skewed such as to involve only one C=O, compared to the symmetric dual-contact that is seen for **7-Ph**.

### Optical and electronic characterization

**Figure 3:**
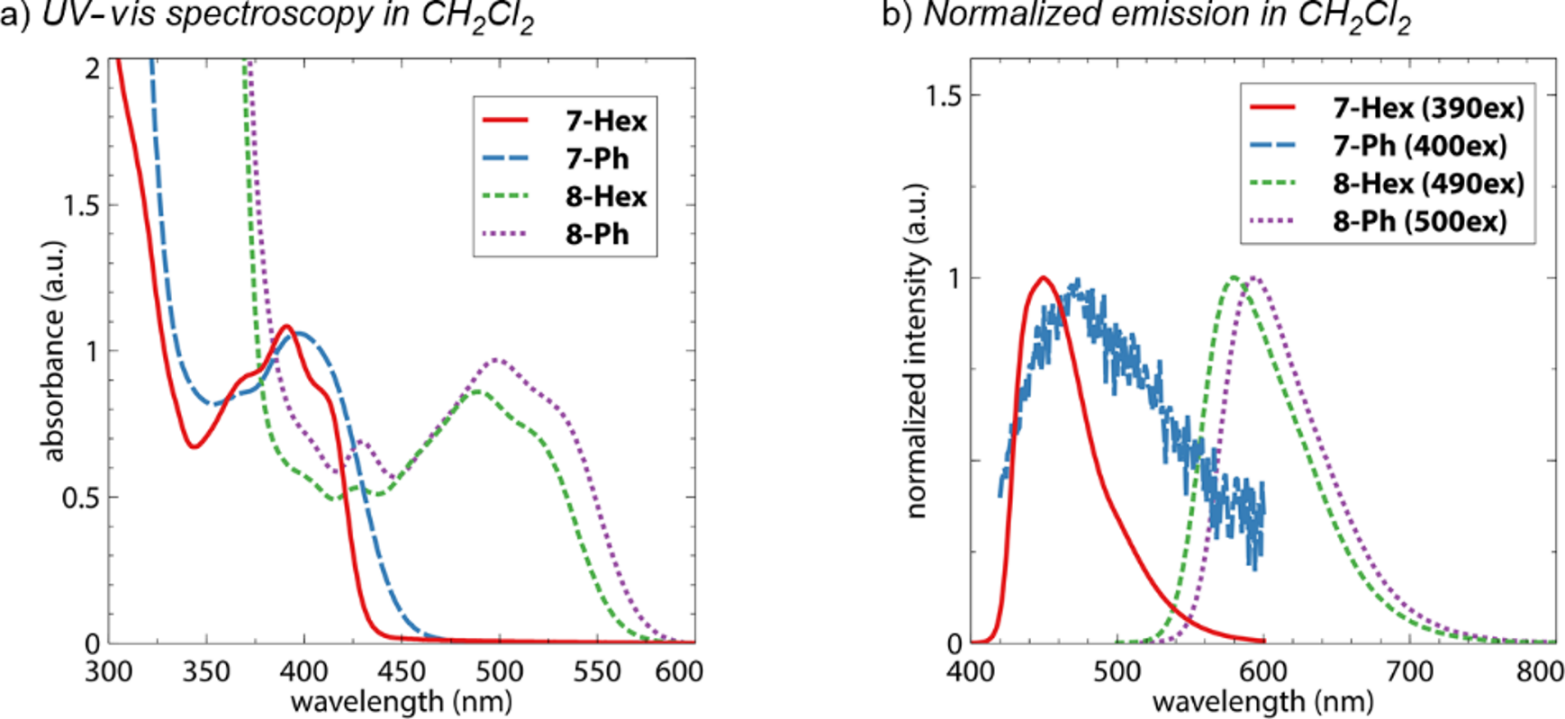
a) Absorption and b) emission spectra of the compounds dissolved in CH_2_Cl_2_.

The absorption spectra of the diimides dissolved in CH_2_Cl_2_ are depicted in Figure 3a. All of these compounds exhibit broad absorption bands. **7-Hex** has more well-defined features with λ_max_ = 391 nm while **7-Ph** has a slightly longer wavelength absorption with λ_max_ = 398 nm. A similar trend is observed for **8-Hex** and **8-Ph**, with λ_max_ = 489 and 499 nm, respectively. These absorption features are roughly comparable to other naphthalene and anthracene diimides that have been reported in the literature. The emission profiles of all four compounds are shown in Figure 3b. While *N*,*N*’-dibutyl-1,4,5,8-naphthalene diimide has low fluorescence intensity (Φ = 0.006) [20], **7-Hex** emits more efficiently with Φ = 0.41. Interestingly, **7-Ph** has nearly no emission intensity, as evidenced by the low signal-to-noise ratio in the data and a near-zero quantum yield when excited at 400 nm. This fluorescence quenching is likely related to non-radiative emission that is observed for *N*-phenyl-substituted imides [21]. It is possible this effect is more significant for **7-Ph** than **8-Ph** because the naphthalene core is less conformationally locked than the anthracene scaffold.

**Figure 4:**
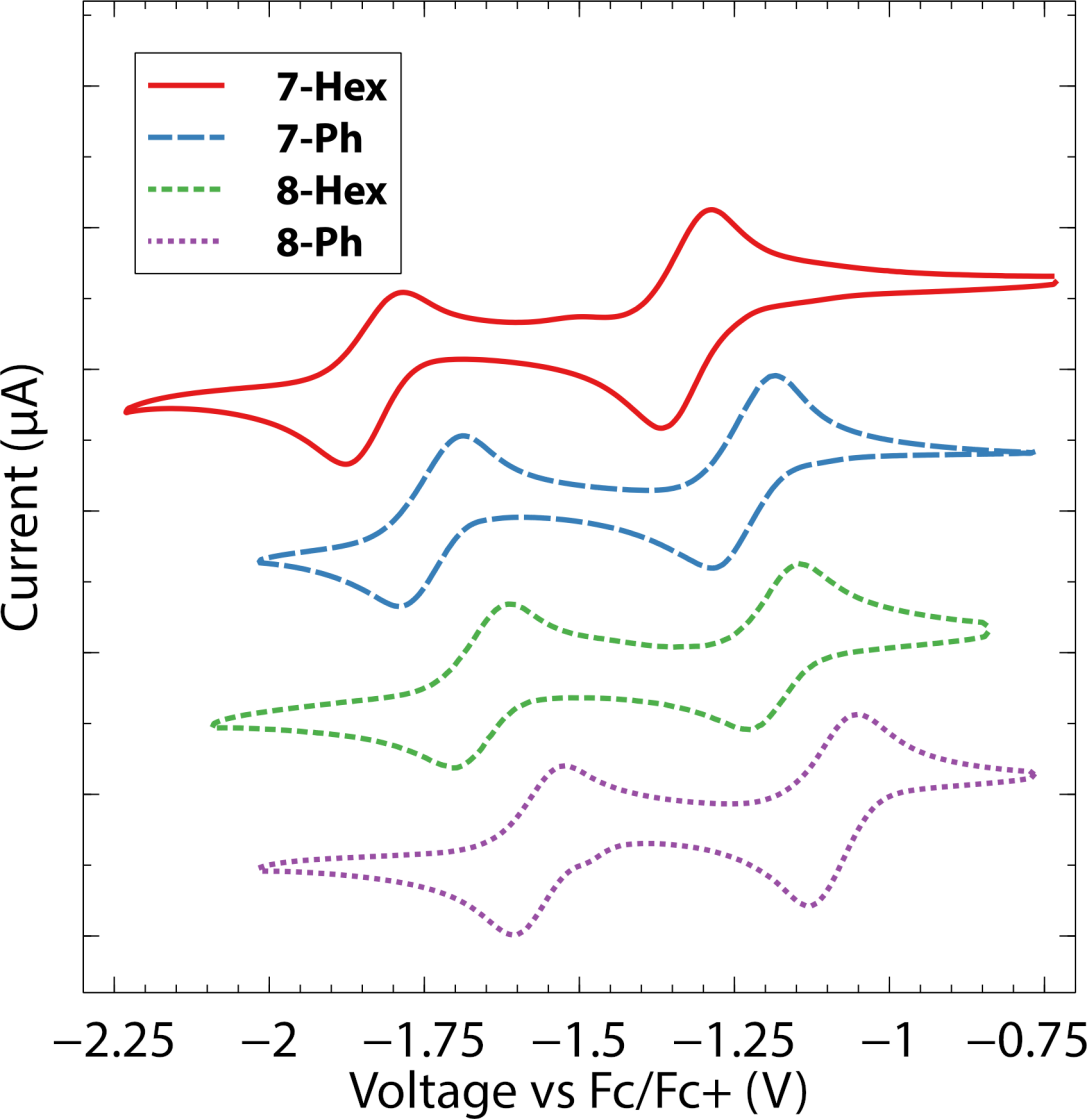
Cyclic voltammograms of the compounds collected on ca*.* 1 mM solutions of the analyte in CH_2_Cl_2_ with 0.1 M Bu_4_NPF_6_ as electrolyte. The major *y*-axis tick mark spacing corresponds to 5 μA.

As is expected for aromatic diimides, the title compounds undergo reversible chemical reduction processes, as determined by cyclic voltammetry in CH_2_Cl_2_ solvent (Figure 4). There are two factors at play. The imide substitution is impactful as the *N*-phenyl derivatives are roughly by 100 mV easier to reduce than the *N*-hexyl analogs. The anthracene scaffold also lends itself to a more facile reduction process, with an approximately 150 mV shift of the event toward more positive potentials for **8-R** vs **7-R**. When compared to other structural isomers, aromatic diimides with 5-membered cyclic imides tend to be slightly harder to reduce than those with 6-membered cyclic imides (Table 1). Overall, however, the *cata*-annulation does not lead to substantially different electrochemical behavior, which is encouraging because we had anticipated that the deflection away from planarity caused by adjacent placement of cyclic imides might adversely affect extent of π-delocalization.

**Table 1:** Summary and comparison of optical and electronic properties.

	*E*_½_ (V vs Fc/Fc^+^)^a^							
	*E*_2r_ (V)	*E*_1r_ (V)	λ_max_^b^ (nm)	λ_em_ (nm)	*E*_g_^c^ (eV)	LUMO^d^ (eV)	HOMO^e^ (eV)	Φ

**7-Hex**	−1.83	−1.33	391	450	2.82	−3.5	−6.3	0.41
**7-Ph**	−1.74	−1.23	398	519^f^	2.64	−3.6	−6.2	<0.01
1,2,5,6-NDI [9]		−1.20^g^				−3.6		
1,4,5,8-NDI [22]	−1.51	−1.10	370		3.18	−3.7	−6.9	0.006 [20]
**8-Hex**	−1.66	−1.19	489	575	2.15	−3.6	−5.7	0.20
**8-Ph**	−1.56	−1.09	498	595	2.07	−3.5	−5.6	0.04
1,9,5,10-ADI^h^ [14]	−1.40^g^	−1.10^g^	480	525	2.2	−3.8	−6.0	
2,3,6,7-ADI [13]		−1.69						
**9** [2]	−1.30	−0.76	404		2.56	−4.2	−6.8	

^a^Unless otherwise noted, ca. 1 mM analyte in CH_2_Cl_2_, 0.1 M Bu_4_NPF_6_. ^b^Unless otherwise noted, longest wavelength absorption maxima of analyte in CH_2_Cl_2_ solution. *^c^*Optical bandgap estimated from absorption onset. ^d^LUMO estimated from reduction onset. ^e^HOMO = LUMO − *E*_g_. ^f^Low intensity emission. ^g^Estimated from graphical data. ^h^PhCl solvent.

**Figure 5:**
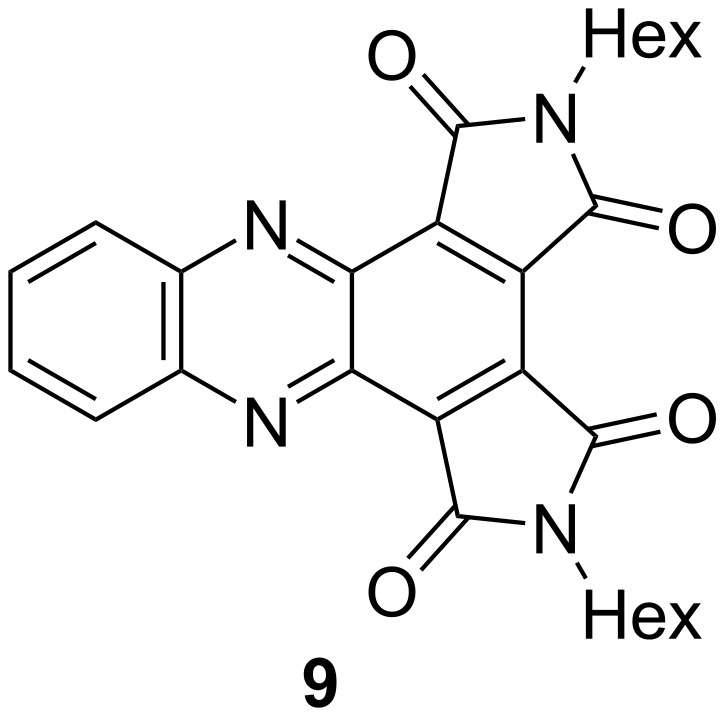
Structural formula of **9**, the diaza-analog of compound **8-Hex** that was reported previously [2].

As part of our previous work constructing heteroacenes bearing *cata*-imide groups, we investigated the 9,10-diaza analog of compound **8-Hex** (**9**, Figure 5). It is interesting to note that the all-carbon scaffold in **8-Hex** results in a narrower bandgap than that of **9**, with Δλ_max_ = 85 nm. This difference can be attributed to a significantly higher HOMO level in **8-Hex** arising from having fewer electronegative atoms in the aromatic backbone. For the same reasons, compound **9** is a superior electron acceptor by 0.36 V. These trends confirm the value of backbone atom substitution for fine-tuning molecular properties.

## Conclusion

In conclusion, we have demonstrated the facile construction of two new aromatic diimide scaffolds: 1,2,3,4-naphthalene and -anthracene diimides through a cycloaddition approach to construct the aromatic backbone prior to imide formation. The physical characterization of the title compounds indicates that they are optically and electronically similar to previously reported naphthalene and anthracene diimides, absorbing/emitting light in the visible region and readily undergoing one-electron-reduction processes. As such, this work opens the possibility of incorporating the 1,2,3,4-naphthalene and -anthracene diimide motifs as productive building blocks in imide-based organic materials.

## Supporting Information

File 1 Experimental procedures, synthetic protocols, and X-ray crystallographic data.

File 2 Crystallographic information file for compound **7-Ph**.

File 3 Crystallographic information file for compound **8-Ph**.

## Data Availability

All data that supports the findings of this study is available in the published article and/or the supporting information to this article.
