# Supplementary material for: Synthesis and characterization of 1,2,3,4-naphthalene and anthracene diimides
Source: Beilstein J Org Chem. 2024 Jul 25;20:1767–72. doi: 10.3762/bjoc.20.155 (PMC11285063; doi:10.3762/bjoc.20.155)
Supplement: File 1 — Experimental procedures, synthetic protocols, and X-ray crystallographic data. [file Beilstein_J_Org_Chem-20-1767-s001.pdf]

BEILSTEIN JOURNAL OF ORGANIC CHEMISTRY

Supporting Information

for

Synthesis and characterization of 1,2,3,4-naphthalene and anthracene diimides

Adam D. Bass, Daniela Castellanos, Xavier A. Calicdan and Dennis D. Cao

Beilstein J. Org. Chem. **2024,** *20,* 1767–1772. doi:10.3762/bjoc.20.155

Experimental procedures, synthetic protocols, and X-ray crystallographic data

License and Terms: This is a supporting information file under the terms of the Creative Commons Attribution License (https://creativecommons.org/licenses/by/4.0). Please note that the reuse, redistribution and reproduction in particular requires that the author(s) and source are credited and that individual graphics may be subject to special legal provisions.
The license is subject to the *Beilstein Journal of Organic Chemistry* terms and conditions: (https://www.beilstein-journals.org/bjoc/terms)

Table of contents

S1 General experimental methods S2

S2 Synthetic procedures and NMR data S2

S3 X-ray crystallography S14

S3.1 Crystallography of **7-Ph** S14

S3.2 Crystallography of **8-Ph** S17

S3.3 Crystallography references S20

S1 General experimental methods

Reagents were purchased from TCI Chemicals or Sigma-Aldrich and used as supplied. Anhydrous solvents were prepared by drying commercial solvents over 3 or 4 Å molecular sieves or obtained from a Pure Process Tech dry solvent system. Molecular sieves were rendered anhydrous by dehydration in a conventional kitchen microwave oven (3 × 1 min irradiation cycles). ^{1}H NMR and ^{13}C NMR spectra were collected on a Bruker Avance III 400 MHz and referenced to residual solvent as the internal standard ($CDCl_3$: ^{1}H 7.26 ppm and ^{13}C 77.16 ppm, DMSO-d_6: ^{1}H 2.50 ppm and ^{13}C 39.52 ppm; CD_2Cl_2: ^{1}H 5.32 ppm). X-ray analysis was performed on a Bruker-AXS D8 Venture diffractometer purchased through a grant from NSF/MRI (#1229400) and the University of Minnesota. UV–vis data were collected with an Evolution™ 300 UV–vis Spectrophotometer. Fluorescence data were collected with a Jasco FP-8550ST spectrofluorometer. Absolute internal quantum yields (ϕ) were measured using an integrating sphere. Cyclic voltammetry experiments were performed using a Pine Research WaveNow potentiostat equipped with a Pt working electrode (1.6 mm diameter disk, PCTFE shroud), Pt coil counter electrode (PCTFE shroud), and non-aqueous reference electrode (Ag wire, 0.01 M $AgNO_3$, 0.1 M Bu_4PF_6, MeCN). Electrochemical samples (ca. 1.0 mM analyte, 0.1 M Bu_4PF_6) were deoxygenated by bubbling Ar through the solutions. Ferrocene (Fc) was used as an internal standard and each sample was evaluated both with and without Fc to ascertain that there were no significant interactions between Fc and the analyte.

S2 Synthetic procedures

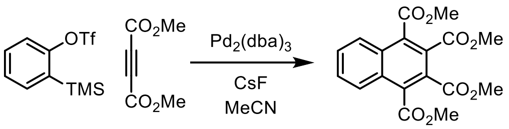

Figure S1. Synthesis of **3**.

<u>Procedure:</u> An oven-dried 250 mL Schlenk tube under Ar was charged with a stirring bar, $Pd_2(dba)_3$ (325 mg, 355 μmol, 0.05 equiv), and oven-dried CsF (2.16 g, 14.2 mmol, 2.0 equiv). The Schlenk tube was capped and subjected to vacuum–Ar backfill (3 cycles). Subsequently, anhydrous MeCN (142 mL), dimethyl acetylenedicarboxylate (4.35 mL, 35.5 mmol, 5 equiv), and 2-(trimethylsilyl)phenyl triflate (1.72 mL, 7.11 mmol, 1.0 equiv) were added by syringe. The reaction mixture was stirred under positive Ar pressure at room temperature for 18 hours.

<u>Workup:</u> The crude reaction mixture was mixed with Celite in a 500 mL round-bottomed flask and subjected to rotary evaporation to remove volatile substances.

<u>Purification:</u> The crude Celite mixture was purified by column chromatography over SiO_2 using 60/40 hexanes/EtOac (v/v) as eluent, yielding **3** as an off-white powder (1.59 g, 4.41 mmol, 62% yield) that exhibits blue fluorescence.

<u>Characterization:</u> **^{1}H NMR** (400 MHz, $CDCl_3$) δ 8.11–8.04 (m, 2H), 7.75–7.68 (m, 2H), 4.02 (s, 6H), 3.92 (s, 6H). The data matches the characterization of compound **25a** in *J. Org. Chem.* **2000**, 65, 6944.

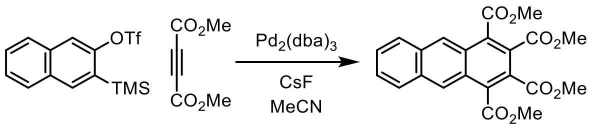

Figure S2. Synthesis of **4**.

Procedure: An oven-dried 250 mL Schlenk tube under Ar was charged with a stirring bar, $Pd_2(dba)_3$ (263 mg, 287 μmol, 0.05 equiv), and oven-dried CsF (1.74 g, 11.5 mmol, 2.0 equiv). The Schlenk tube was capped and subjected to vacuum–Ar backfill (3 cycles). Subsequently, anhydrous MeCN (115 mL), dimethyl acetylenedicarboxylate (3.52 mL, 28.7 mmol, 5 equiv), and 3-(trimethylsilyl)naphthalene-2-yl triflate (1.59 mL, 5.74 mmol, 1.0 equiv) were added by syringe. The reaction mixture was stirred under positive Ar pressure at room temperature for 18 hours.

Workup: The crude reaction mixture was mixed with Celite in a 500 mL round-bottomed flask and subjected to rotary evaporation to remove volatile substances.

Purification: The crude Celite mixture was purified by column chromatography over SiO_2 using 60/40 hexanes/EtOac (v/v) as eluent, yielding **4** as a yellow solid (1.14 g, 2.78 mmol, 48% yield) that exhibits orange fluorescence in solution. Note: the product contains a small amount of byproduct believed to originate from the polymerization of dimethyl acetylenedicarboxylate. This impurity is removed by purification after subsequent reactions.

Characterization: **^{1}H NMR** (400 MHz, $CDCl_3$) δ 8.63 (s, 2H), 8.06–7.97 (m, 2H), 7.62–7.53 (m, 2H), 4.09 (s, 6H), 3.94 (s, 6H). **^{13}C NMR** (101 MHz, $CDCl_3$) δ 167.55, 166.86, 134.73, 133.39, 128.74, 127.97, 126.79, 126.46, 126.27, 53.36, 53.26.

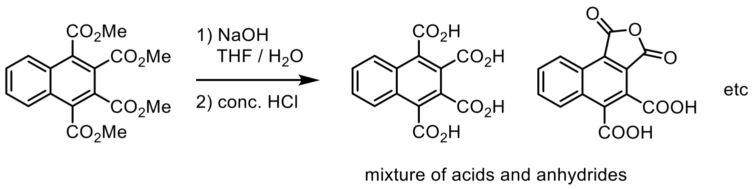

Figure S3. Synthesis of **5**.

Procedure: A round-bottomed flask was charged with a stirring bar, tetraester **3** (1.56 g, 4.33 mmol, 1.0 equiv), THF (50 mL), and H_2O (150 mL). Once **3** was mostly dissolved, NaOH (3.46 g, 86.6 mmol, 20.0 equiv) was added to the flask and the reaction mixture was stirred at room temperature for 18 hours.

Workup: Upon confirmation of reaction completion by TLC, the volatiles were removed by rotary evaporation. The crude residue was then acidified with conc. HCl, with additions performed until the resulting solution was pH < 2. In a fume hood, the mixture was concentrated to dryness by rotary evaporation. The solid residue was then dispersed in acetone (100 mL) and stirred for 30 minutes, after which the undissolved inorganic salts were removed by gravity filtration. Evaporation of the filtration yielded a brown solid (1.28 g) that was found to be **5**, a mixture of acid and anhydride forms (see Figure S13).

Purification: None. The crude mixture was used without further purification.

Characterization: See NMR spectra.

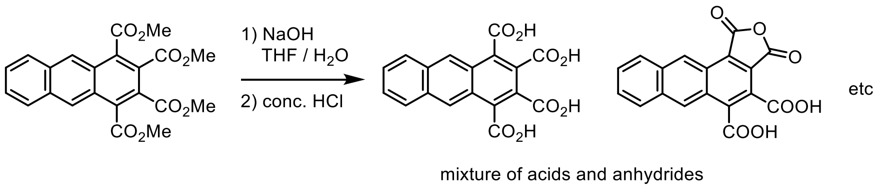

Figure S5. Synthesis of **6**.

Procedure: A round-bottomed flask was charged with a stirring bar, tetraester **2** (30 mg, 73.1 μmol, 1.0 equiv), THF (4 mL), and H_2O (1 mL). Once **2** was somewhat dissolved, NaOH (58.5 mg, 1.46 mmol, 20.0 equiv) was added to the flask and the reaction mixture was stirred at room temperature for 18 hours.

Workup: Upon confirmation of reaction completion by TLC, the volatiles were removed by rotary evaporation. The crude residue was then acidified with conc. HCl, with additions performed until the resulting solution was pH < 2. In a fume hood, the mixture was concentrated to dryness by rotary evaporation. The solid residue was then dispersed in acetone (50 mL) and stirred for 30 minutes, after which the undissolved inorganic salts were removed by gravity filtration. Evaporation of the filtration yielded a red solid (21.8 mg) that was found to be **4**, a mixture of acid and anhydride forms (see Figure S13).

Purification: None. The crude mixture was used without further purification.

Characterization: See NMR spectra.

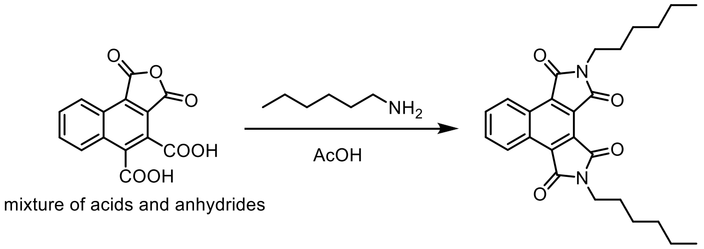

Figure S7. Synthesis of **7-Hex**.

Procedure: A round-bottomed flask was charged with a stirring bar, acid/anhydride mixture **5** (150 mg, 493 μmol, 1.0 equiv assuming 100% tetracarboxylic acid in **5**), and AcOH (16 mL). After stirring for 5 minutes, hexylamine (205 μL, 1.55 mmol, 3.14 equiv) was added by pipette. A reflux condenser was placed on the flask and the reaction mixture was refluxed for 18 hours.

Workup: Upon cooling to room temperature, H_2O (10 mL) was added to the reaction mixture. The resulting precipitate was collected by vacuum filtration and dried overnight to provide a light brown solid.

Purification: The crude was purified by column chromatography over SiO_2 using CH_2Cl_2 as eluent to yield **7-Hex** as a crystalline off-white solid (174 mg, 81% yield).

Characterization: **^{1}H NMR** (400 MHz, $CDCl_3$) δ 9.22 – 9.13 (m, 2H), 7.94 – 7.86 (m, 2H), 3.78 (t, *J* = 7.3 Hz, 4H), 1.74 (p, *J* = 7.4 Hz, 4H), 1.44 – 1.23 (m, 12H), 0.95 – 0.84 (m, 6H). **^{13}C NMR** (101 MHz, $CDCl_3$) δ 168.44, 165.18, 133.22, 132.00, 131.27, 126.03, 125.68, 38.65, 31.50, 28.60, 26.65, 22.61, 14.14.

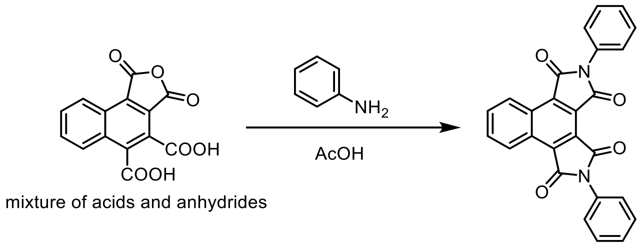

Figure S8. Synthesis of **7-Ph**.

Procedure: A round-bottomed flask was charged with a stirring bar, acid/anhydride mixture **5** (50 mg, 164 μmol, 1.0 equiv assuming 100% tetracarboxylic acid in **5**), and AcOH (5.4 mL). After stirring for 5 minutes, aniline (47 μL, 0.52 mmol, 3.2 equiv) was added by pipette. A reflux condenser was placed on the flask and the reaction mixture was refluxed for 18 hours.

Workup: Upon cooling to room temperature, H_2O (10 mL) was added to the reaction mixture. The resulting precipitate was collected by vacuum filtration and dried overnight to provide a dark brown solid.

Purification: The crude was dissolved in a minimum volume of CH_2Cl_2 and after one day of slow evaporation of the solution, the resulting yellow solid was collected by vacuum filtration and found to be **7-Ph** (28 mg, 41% yield).

Characterization: **^{1}H NMR** (400 MHz, DMSO-*d*$_6$) δ 9.20 – 9.13 (m, 2H), 8.14 – 8.07 (m, 2H), 7.58 – 7.46 (m, 10H). **^{13}C NMR** (101 MHz, DMSO-*d*$_6$) δ 167.30, 163.82, 133.00, 132.42, 131.73, 130.58, 129.15, 128.55, 127.76, 125.71, 125.53

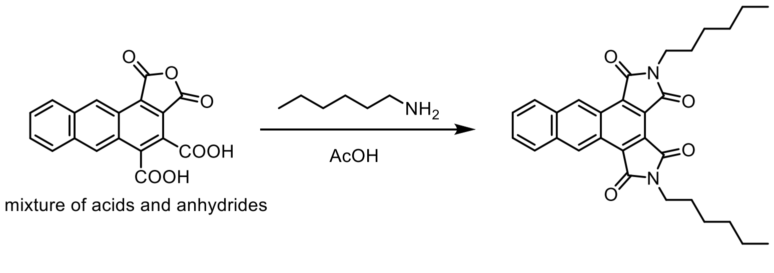

Figure S9. Synthesis of **8-Hex**.

Procedure: A round-bottomed flask was charged with a stirring bar, acid/anhydride mixture **6** (58 mg, 164 μmol, 1.0 equiv assuming 100% tetracarboxylic acid in **6**), and AcOH (16 mL). After stirring for 5 minutes, hexylamine (68 uL, 0.51 mmol, 3.1 equiv) was added by pipette. A reflux condenser was placed on the flask and the reaction mixture was refluxed for 18 hours.

Workup: Upon cooling to room temperature, H_2O (10 mL) was added to the reaction mixture. The resulting precipitate was collected by vacuum filtration and dried overnight to provide a red solid.

Purification: The crude was purified by column chromatography over SiO_2 using CH_2Cl_2 as eluent to yield **8-Hex** as a red solid (59 mg, 74% yield).

Characterization: **^{1}H NMR** (400 MHz, $CDCl_3$) δ 9.60 – 9.55 (m, 2H), 8.12 – 8.03 (m, 2H), 7.69 – 7.60 (m, 2H), 3.83 – 3.74 (m, 4H), 1.76 (p, *J* = 7.3 Hz, 4H), 1.47 – 1.27 (m, 12H), 0.93 – 0.84 (m, 6H). **^{13}C NMR** (101 MHz, $CDCl_3$) δ 168.74, 165.42, 134.46, 133.99, 129.43, 128.85, 126.66, 126.14, 124.84, 38.58, 31.51, 28.65, 26.68, 22.62, 14.15.

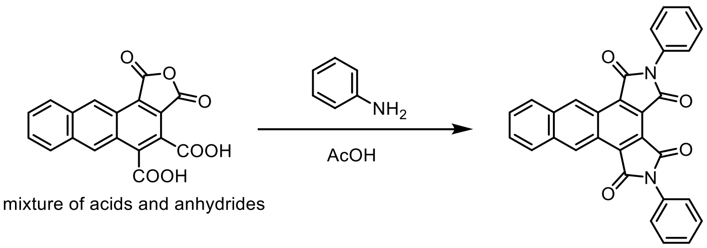

Figure S10. Synthesis of **8-Ph**.

Procedure: A round-bottomed flask was charged with a stirring bar, acid/anhydride mixture **6** (58 mg, 164 μmol, 1.0 equiv assuming 100% tetracarboxylic acid in **6**), and AcOH (5.4 mL). After stirring for 5 minutes, aniline (47 μL, 0.52 mmol, 3.2 equiv) was added by pipette. A reflux condenser was placed on the flask and the reaction mixture was refluxed for 18 hours.

Workup: Upon cooling to room temperature, H_2O (10 mL) was added to the reaction mixture. The resulting precipitate was collected by vacuum filtration and dried overnight to provide a dark red solid.

Purification: The crude was dissolved in a minimum volume of CH_2Cl_2 and allowed to crystallize by a combination of slow evaporation and cooling in a refrigerator. The resulting red solid was collected by vacuum filtration and found to be **8-Ph** (29 mg, 38% yield).

Characterization: **^{1}H NMR** ^{1}H NMR (400 MHz, CD_2Cl_2) δ 9.90 (s, 2H), 8.27 (dd, *J* = 6.5, 3.3 Hz, 2H), 7.76 (dt, *J* = 6.6, 3.3 Hz, 2H), 7.65 – 7.44 (m, 10H). **^{13}C NMR** (101 MHz, DMSO) δ 167.46, 164.01, 133.93, 133.90, 131.74, 129.40, 129.35, 129.16, 128.46, 127.65, 126.36, 125.81, 125.0.

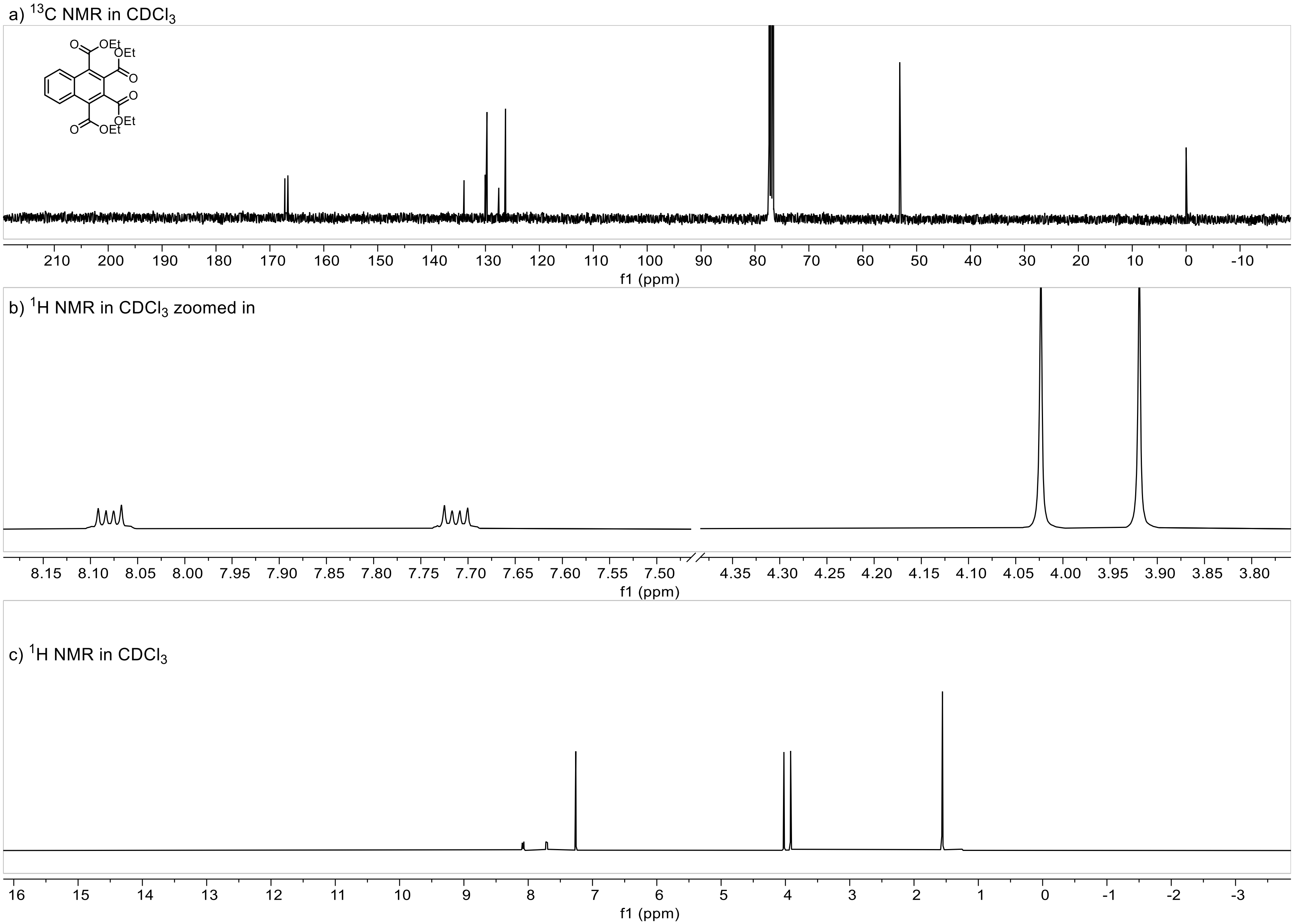

Figure S11. NMR spectra for compound **1**. a) ^{13}C NMR data, b) close view of the relevant subset of ^{1}H NMR data, c) complete ^{1}H NMR data.

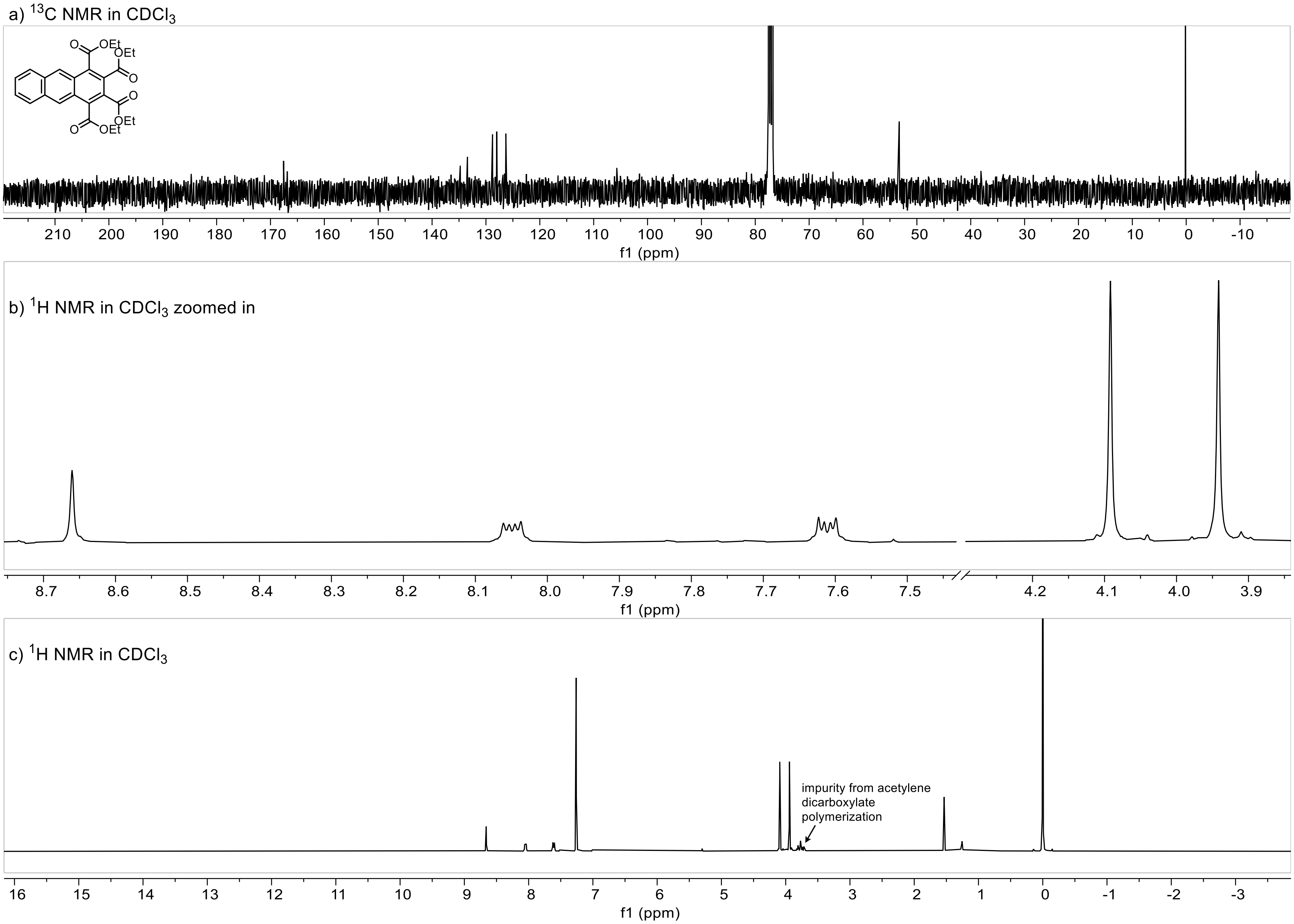

Figure S12. NMR spectra for compound **2**. a) ^{13}C NMR data, b) close view of the relevant subset of ^{1}H NMR data, c) complete ^{1}H NMR data.

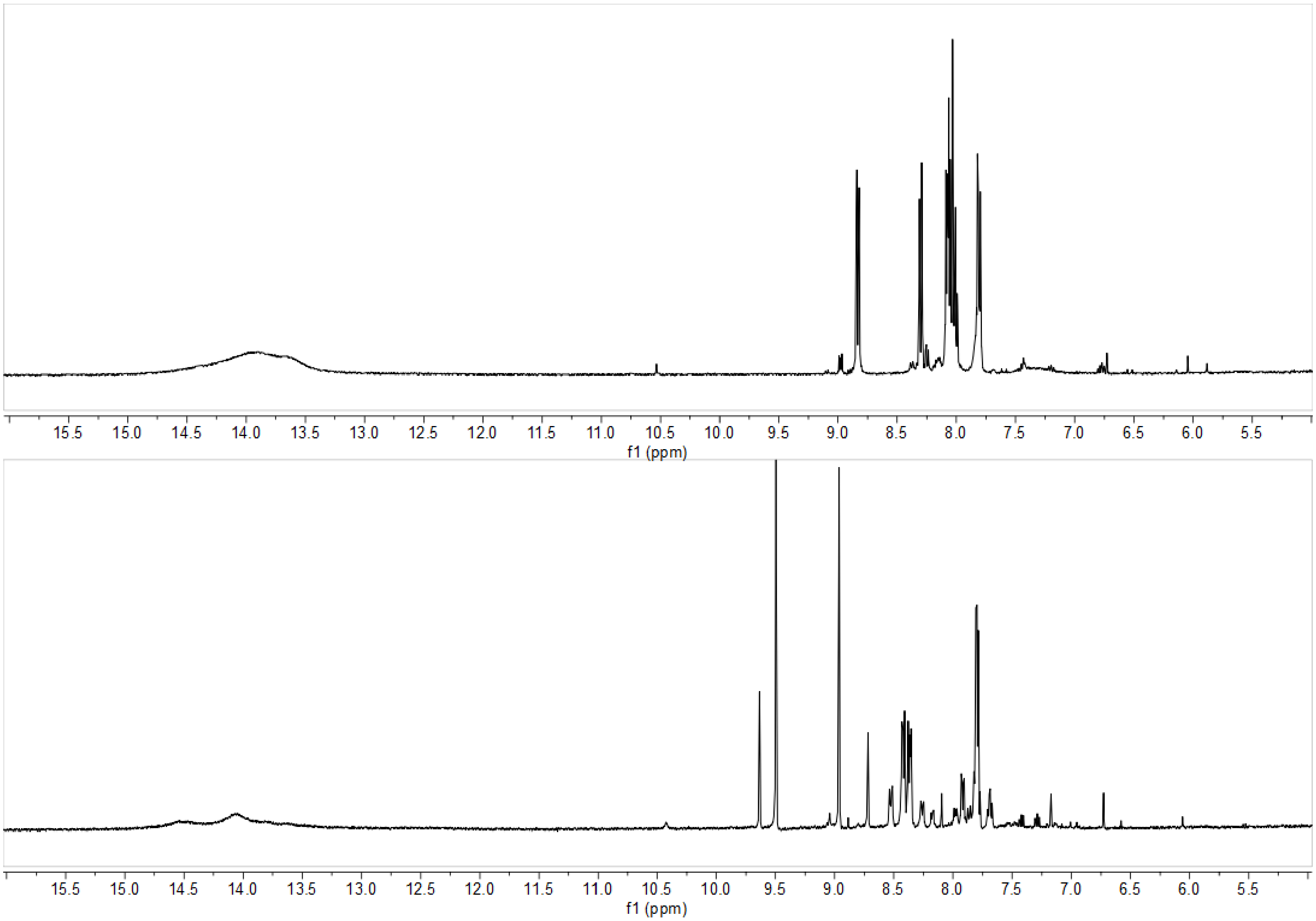

Figure S13. ^{1}H NMR spectrum (DMSO-d_6) showing a complex aromatic / carboxylic acid region suggestive of the formation of a mixture of anhydrides and carboxylic acids for (top) **5** and (bottom) **6.**

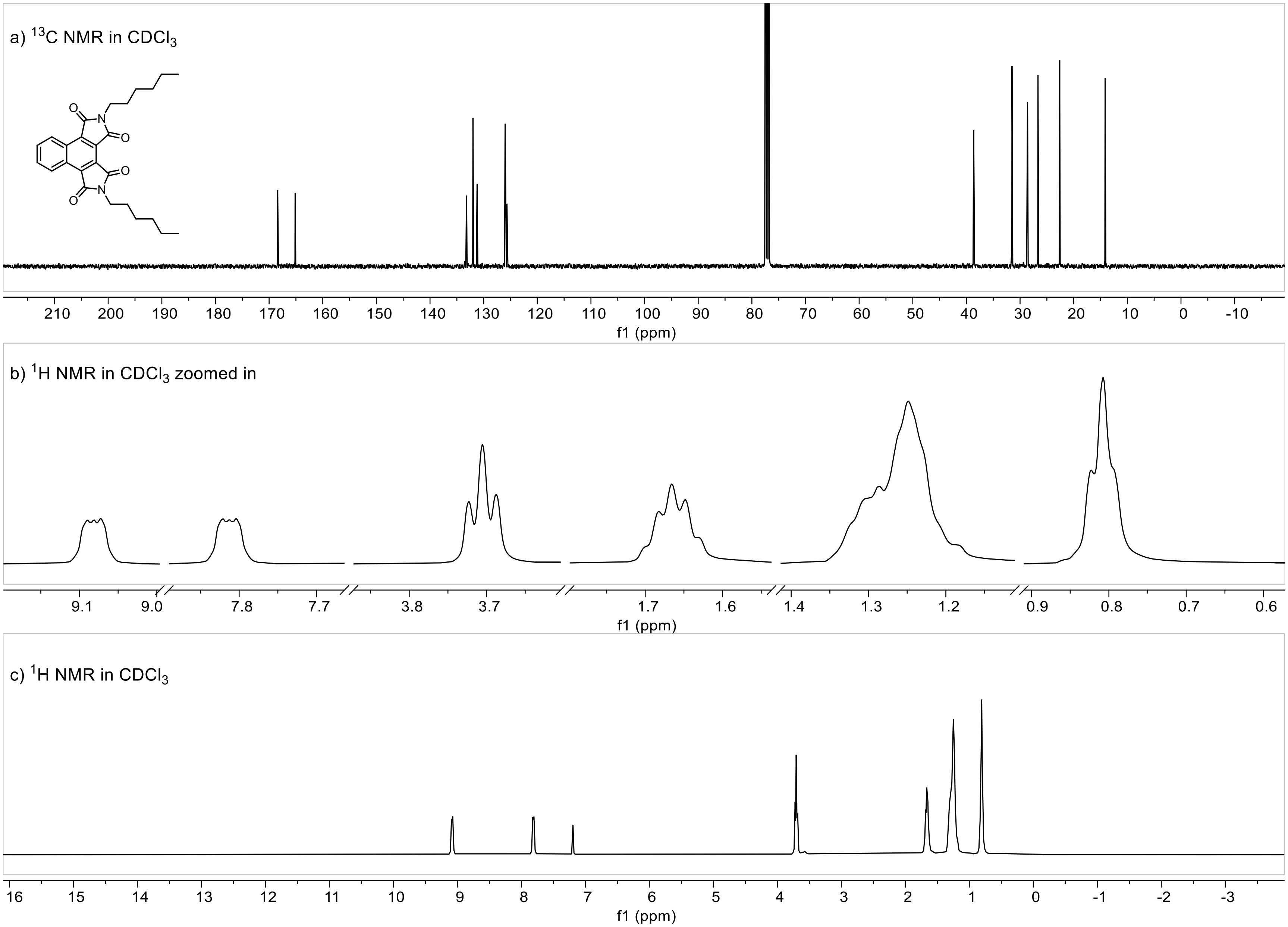

Figure S14. NMR spectra for compound **7-Hex**. a) ^{13}C NMR data, b) close view of the relevant subset of ^{1}H NMR data, c) complete ^{1}H NMR data.

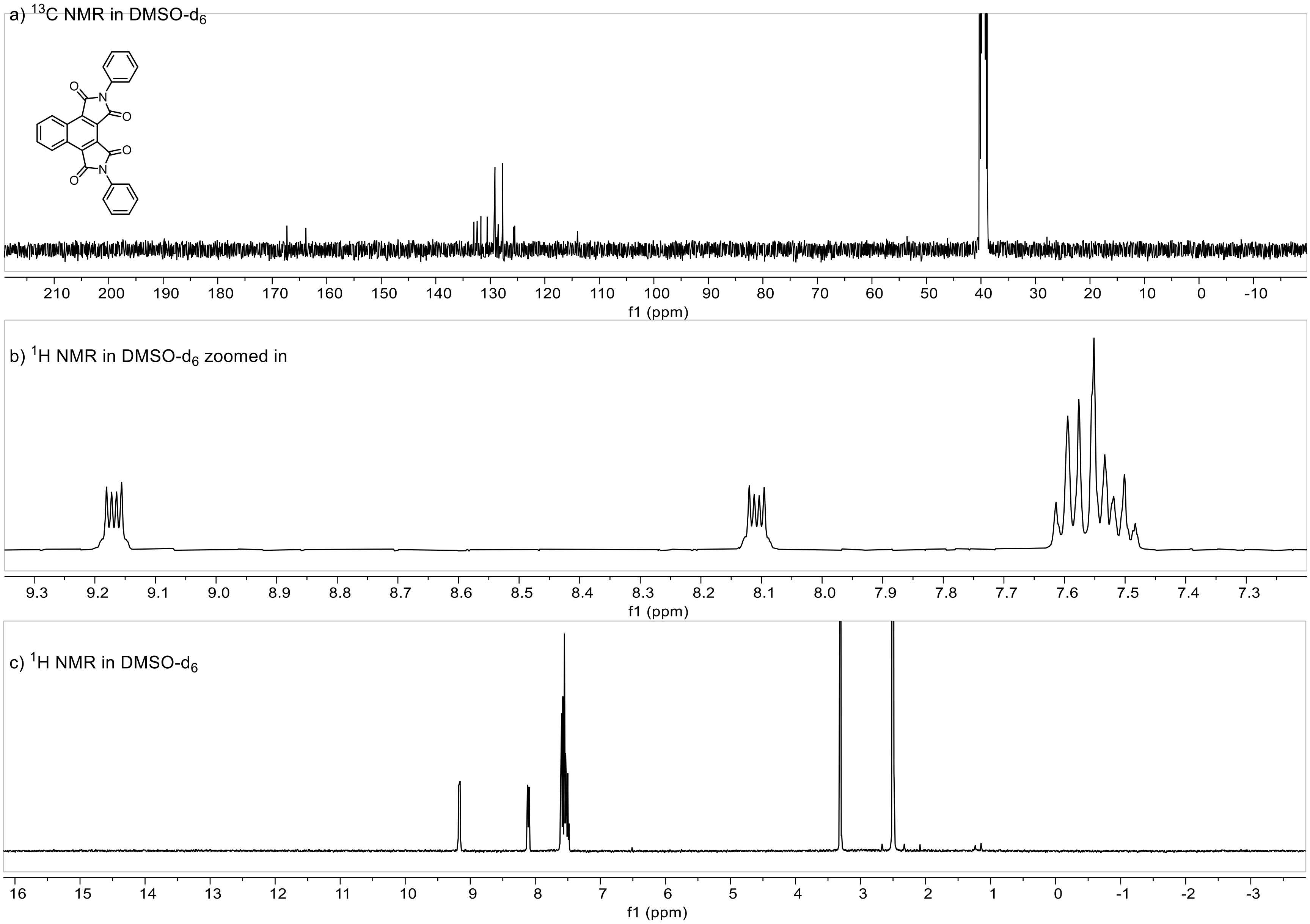

Figure S15. NMR spectra for compound **7-Ph**. a) ^{13}C NMR data, b) close view of the relevant subset of ^{1}H NMR data, c) complete ^{1}H NMR data.

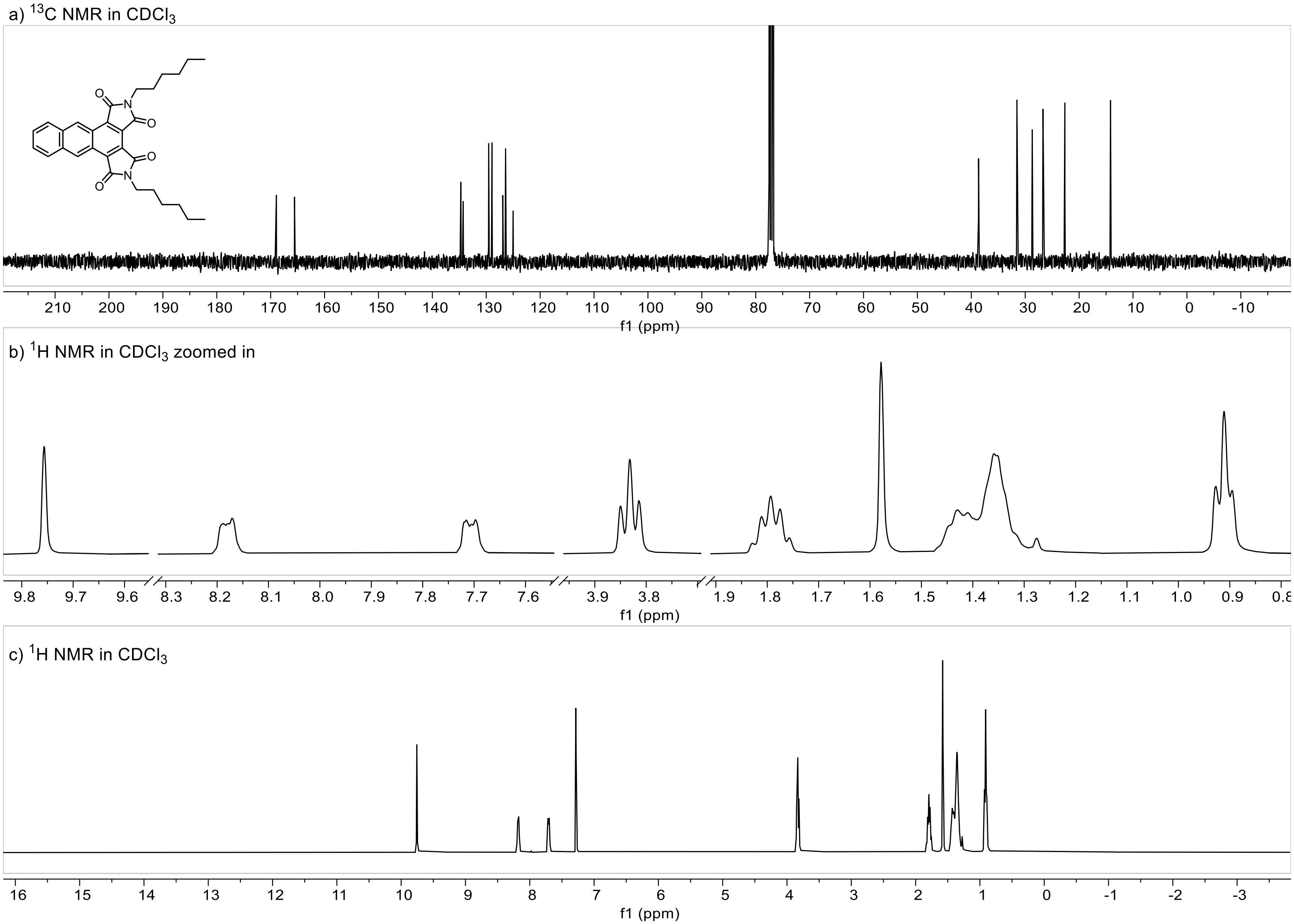

Figure S16. NMR spectra for compound **8-Hex**. a) ^{13}C NMR data, b) close view of the relevant subset of ^{1}H NMR data, c) complete ^{1}H NMR data.

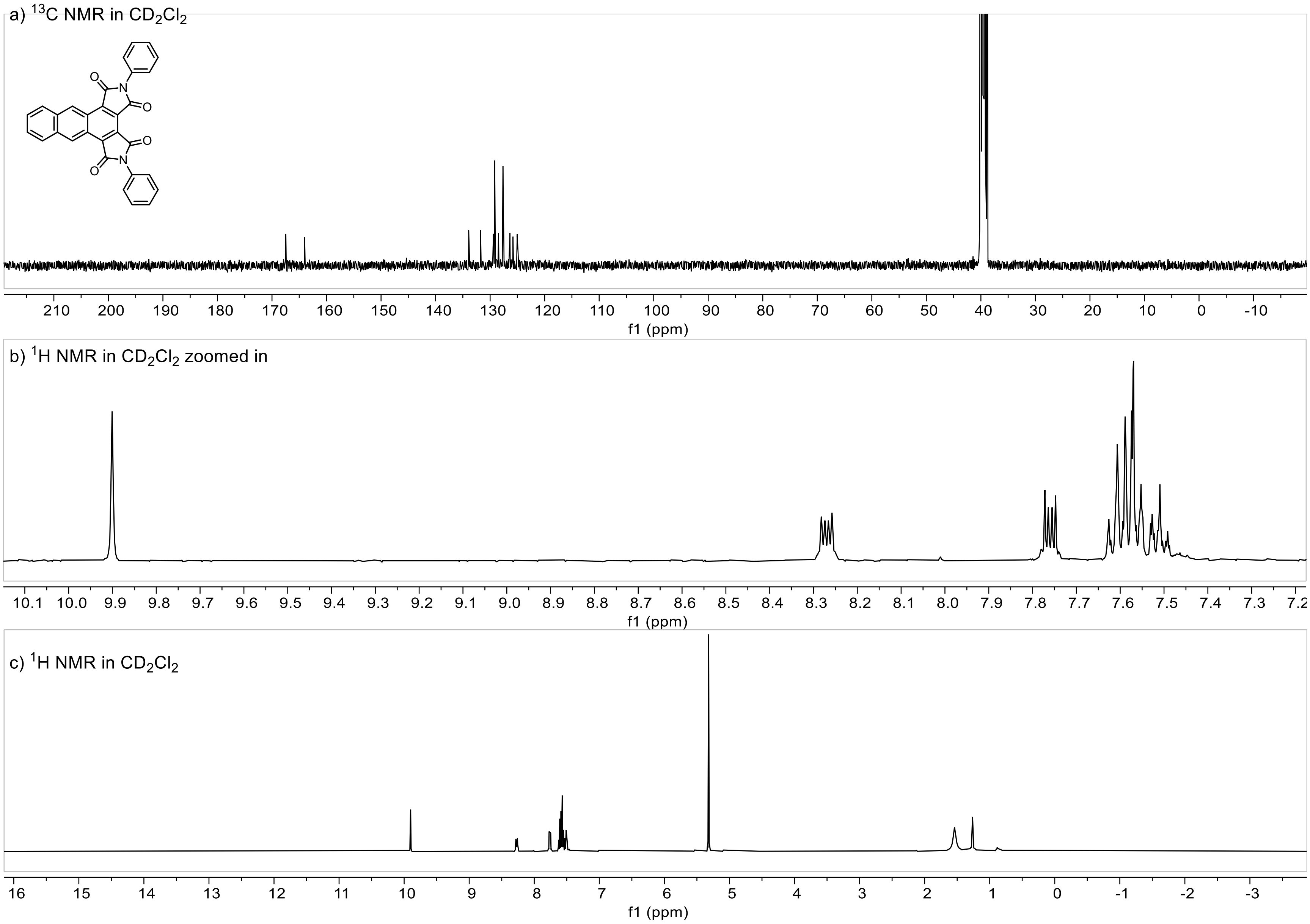

Figure S17. NMR spectra for compound **8-Ph**. a) ^{13}C NMR data, b) close view of the relevant subset of ^{1}H NMR data, c) complete ^{1}H NMR data.

S3. X-ray crystallography

S3.1 Crystallography of 7-Ph

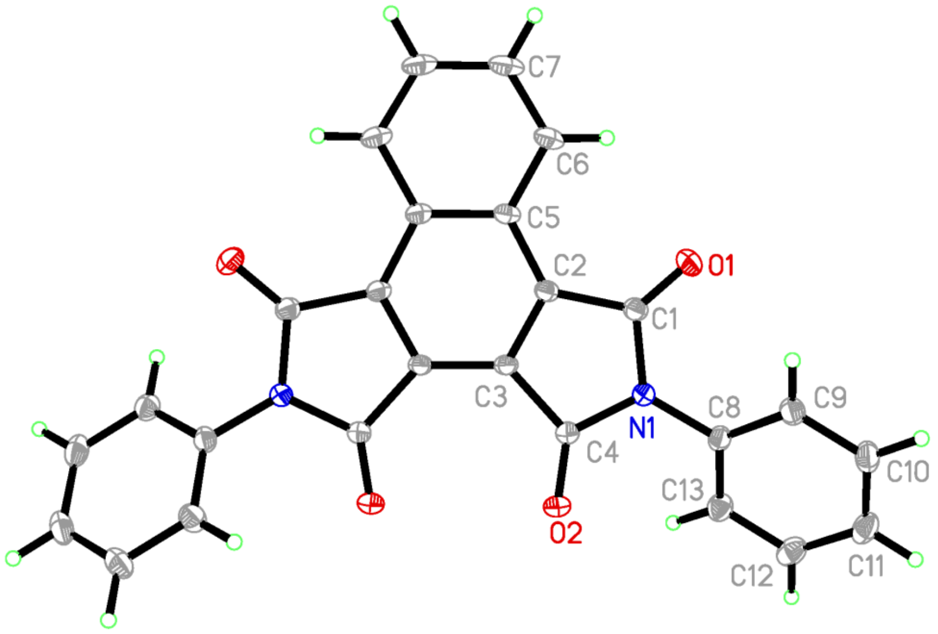

Figure S18. Ellipsoid depiction of a single molecule of **7-Ph** as determined by X-ray crystallography.

Data collection

A crystal (approximate dimensions 0.200 × 0.200 × 0.140 mm^3) was placed onto the tip of a 200 µm diameter MiTeGen Dual-Thickness Microloop and mounted on a Bruker PHOTON-III CPAD diffractometer for a data collection at 130(2) K.[1] A preliminary set of cell constants was calculated from reflections harvested from three sets of frames. These initial sets of frames were oriented such that orthogonal wedges of reciprocal space were surveyed. This produced an initial orientation matrix determined from 908 reflections. The data collection was carried out using MoKα radiation (parabolic mirrors) with a frame time of 5 seconds and a detector distance of 4.0 cm. A strategy program was used to assure complete coverage of all unique data to a resolution of 0.70 Å. All major sections of frames were collected with 1.20° steps in ω or Φ at different detector positions in 2Θ. The intensity data were corrected for absorption and decay (SADABS).[2] Final cell constants were calculated from 2919 strong reflections from the actual data collection after integration (SAINT).[3] Please refer to Table S1 for additional crystal and refinement information.

Structure solution and refinement.

The structure was solved using SHELXT-2018/2 (Sheldrick 2015)4 and refined using SHELXL-2018/3 (Sheldrick 2015).[4] The space group *P*bcn was determined based on systematic absences and intensity statistics. A direct-methods solution was calculated which provided most non-hydrogen atoms from the E-map. Full-matrix least squares / difference Fourier cycles were performed which located the remaining non-hydrogen atoms. All non-hydrogen atoms were

refined with anisotropic displacement parameters. All hydrogen atoms were placed in ideal positions and refined as riding atoms with relative isotropic displacement parameters. The final full matrix least squares refinement converged to $R1$ = 0.0404 and $wR2$ = 0.1071 (F^2, obs. data).

Structure description

The structure is the one suggested. The molecule is located on a crystallographic two-fold axis: Z'=½

Data collection and structure solution were conducted at the X-Ray Crystallographic Laboratory, 192 Kolthoff Hall, Department of Chemistry, University of Minnesota. All calculations were performed using Pentium computers using the current SHELXTL suite of programs.

Table S1. Crystal data and structure refinement for **7-Ph**.

Empirical formula	$C_{26} H_{14} N_2 O_4$	
Formula weight	418.39	
Temperature	130(2) K	
Wavelength	0.71073 Å	
Crystal system	Orthorhombic	
Space group	*P*bcn	
Unit cell dimensions	*a* = 26.4699(18) Å	α = 90°
	b = 10.3060(7) Å	β = 90°
	c = 7.0470(4) Å	γ = 90°
Volume	1922.4(2) $Å^3$	
Z	4	
Density (calculated)	1.446 Mg/m^3	
Absorption coefficient	0.099 mm^{-1}	
F(000)	864	
Crystal color, morphology	yellow , block	
Crystal size	0.200 x 0.200 x 0.140 mm^3	
Theta range for data collection	2.121 to 30.556°	
Index ranges	$-37 \le h \le 30$, $-14 \le k \le 14$, $-10 \le l \le 7$	
Reflections collected	17748	
Independent reflections	2942 [*R*(int) = 0.0342]	
Observed reflections	2570	
Completeness to theta = 25.242°	99.9%	
Absorption correction	Multi-scan	
Max. and min. transmission	0.74547 and 0.4990	
Refinement method	Full-matrix least-squares on F^2	
Data / restraints / parameters	2942 / 0 / 145	
Goodness-of-fit on F^2	1.058	
Final *R* indices [$I > 2\sigma(I)$]	*R*1 = 0.0404, *wR*2 = 0.1071	
R indices (all data)	*R*1 = 0.0458, *wR*2 = 0.1127	
Largest diff. peak and hole	0.428 and –0.284 e $Å^{-3}$	

S3.2 Crystallography of 8-Ph

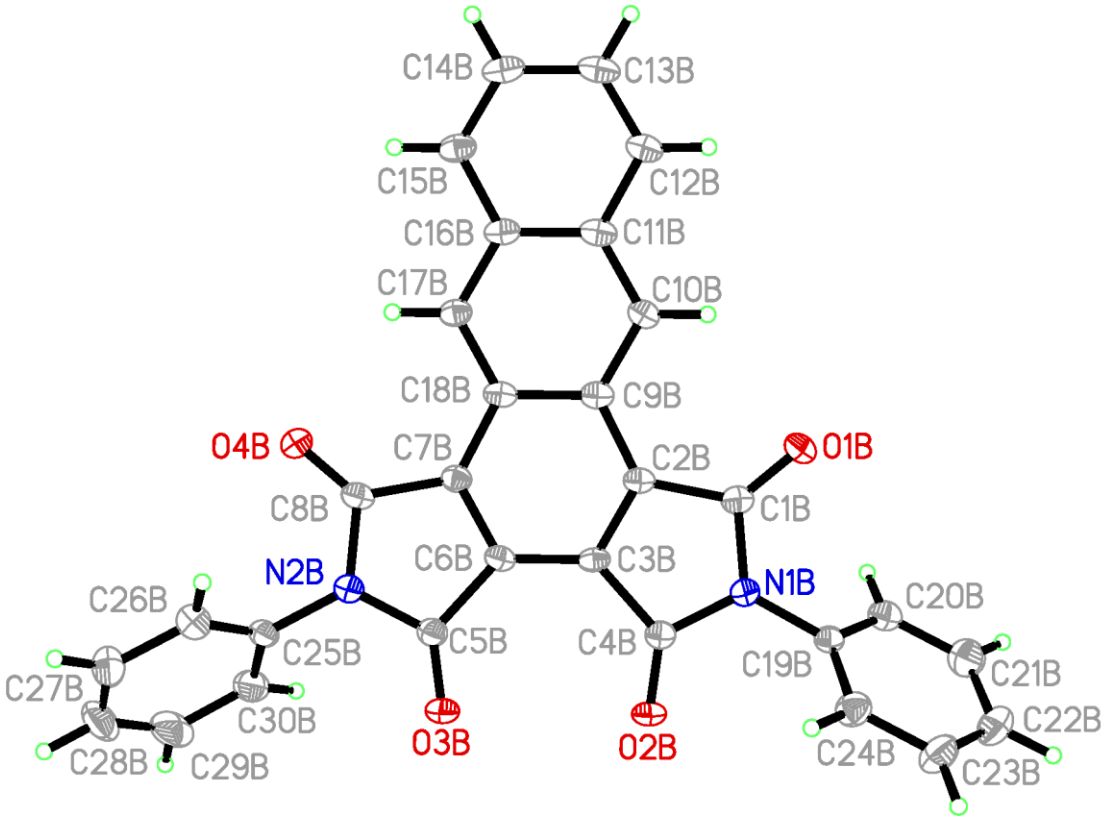

Figure S19. Ellipsoid depiction of a single molecule of **8-Ph** as determined by X-ray crystallography.

Data collection

A crystal (approximate dimensions 0.230 × 0.060 × 0.035 mm^3) was placed onto the tip of a 200 μm diameter MiTeGen Dual-Thickness Microloop and mounted on a Bruker PHOTON-III CPAD diffractometer for a data collection at 130(2) K.[1] A preliminary set of cell constants was calculated from reflections harvested from three sets of frames. These initial sets of frames were oriented such that orthogonal wedges of reciprocal space were surveyed. This produced an initial orientation matrix determined from 541 reflections. The data collection was carried out using MoKα radiation (parabolic mirrors) with a frame time of 100 seconds and a detector distance of 10.5 cm. A strategy program was used to assure complete coverage of all unique data to a resolution of 0.80 Å. All major sections of frames were collected with 1.00° steps in ω or Φ at different detector positions in 2Θ. The intensity data were corrected for absorption and decay (SADABS).[2] Final cell constants were calculated from 2995 strong reflections from the actual data collection after integration (SAINT).[3] Please refer to Table S2 for additional crystal and refinement information.

Structure Solution and Refinement.

The structure was solved using SHELXT-2018/2 (Sheldrick 2015)4 and refined using SHELXL-2018/3 (Sheldrick 2015).[4] The space group *P*bca was determined based on systematic absences and intensity statistics. A direct-methods solution was calculated which provided most non-

hydrogen atoms from the E-map. Full-matrix least squares / difference Fourier cycles were performed which located the remaining non-hydrogen atoms. All non-hydrogen atoms were refined with anisotropic displacement parameters. All hydrogen atoms were placed in ideal positions and refined as riding atoms with relative isotropic displacement parameters. The final full matrix least squares refinement converged to $R1$ = 0.0631 and $wR2$ = 0.1457 (F^2, obs. data).

Structure description

The structure is the one suggested. There are two molecules of interest along with one DCM solvate. The specimen used was small so 100 second frame times were used.

Data collection and structure solution were conducted at the X-Ray Crystallographic Laboratory, 192 Kolthoff Hall, Department of Chemistry, University of Minnesota. All calculations were performed using Pentium computers using the current SHELXTL suite of programs.

Table S2. Crystal data and structure refinement for **8-Ph**.

Empirical formula	$C_{61} H_{34} Cl_2 N_4 O_8$	
Formula weight	1021.82	
Temperature	130(2) K	
Wavelength	0.71073 Å	
Crystal system	Monoclinic	
Space group	*P*bca	
Unit cell dimensions	*a* = 12.8560(9) Å	α = 90°
	b = 13.2882(10) Å	β = 90°
	c = 54.898(4) Å	γ = 90°
Volume	9378.3(12) $Å^3$	
Z	8	
Density (calculated)	1.447 Mg/m^3	
Absorption coefficient	0.206 mm^{-1}	
F(000)	4208	
Crystal color, morphology	red, needle	
Crystal size	0.230 x 0.060 x 0.035 mm^3	
Theta range for data collection	2.226 to 26.231°	
Index ranges	$-11 \le h \le 15$, $-15 \le k \le 16$, $-53 \le l \le 68$	
Reflections collected	63324	
Independent reflections	9387 [*R*(int) = 0.0661]	
Observed reflections	6975	
Completeness to theta = 25.242°	99.8%	
Absorption correction	Multi-scan	
Max. and min. transmission	0.7454 and 0.6212	
Refinement method	Full-matrix least-squares on F^2	
Data / restraints / parameters	9387 / 0 / 676	
Goodness-of-fit on F^2	1.076	
Final *R* indices [I>2σ(I)]	*R*1 = 0.0631, *wR*2 = 0.1457	
R indices (all data)	*R*1 = 0.0914, *wR*2 = 0.1577	
Largest diff. peak and hole	0.332 and –0.727 e $Å^{-3}$	

S3.3 Crystallography references

[1]APEX3, Bruker Analytical X-ray Systems, Madison, WI (2014).

[2]SADABS, Bruker Analytical X-ray Systems, Madison, WI (2014).

[3]SAINT Bruker Analytical X-ray Systems, Madison, WI (2014).

[4]SHELXTL 2014, Bruker Analytical X-Ray Systems, Madison, WI (2013); G. M. Sheldrick, *Acta Cryst.* **A64**, 112-122 (2008).
